# 2-(4-Fluoro­phen­yl)-4-(4-meth­oxy­phen­yl)-5-(piperidin-1-ylmeth­yl)thia­zole

**DOI:** 10.1107/S1600536810054103

**Published:** 2011-01-12

**Authors:** Chang-Bin Guo, Chao Lv, Wei Wei, Hua Zhou

**Affiliations:** a Department of Chemistry, Capital Normal University, Beijing 100048, People’s Republic of China

## Abstract

In the title compound, C_22_H_23_FN_2_OS, the piperidine ring shows chair confirmation and the two benzene rings make a dihedral angle of 17.0 (6)°. The thia­zole fragment is essentially planar with an r.m.s. deviation of 0.004 (2) Å and a maximum deviation of 0.006 (2) Å.. In the crystal, inter­molecular C—H⋯π inter­actions lead to the formation of a layer structure.

## Related literature

For the biological activity of thia­zole derivatives, see: Guo *et al.* (2006 ▶); Karegoudar *et al.* (2008 ▶) Reddy *et al.* (1999 ▶);. For related structures, see: Mitsutaka *et al.* (2006 ▶); Takayuki *et al.* (2009 ▶).
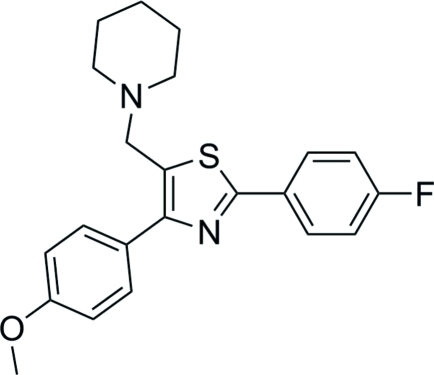


## Experimental

### 

#### Crystal data

C_22_H_23_FN_2_OS
*M* *_r_* = 382.48
Triclinic, 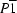

*a* = 10.7565 (2) Å
*b* = 10.8846 (2) Å
*c* = 11.0179 (2) Å
α = 67.035 (1)°
β = 63.881 (1)°
γ = 60.768 (1)°
*V* = 985.16 (3) Å^3^
*Z* = 2
Mo *K*α radiation
μ = 0.19 mm^−1^
*T* = 296 K
0.26 × 0.26 × 0.24 mm

#### Data collection

Bruker APEXII CCD area-detector diffractometer
Absorption correction: multi-scan (*SADABS*; Bruker, 2007 ▶) *T*_min_ = 0.659, *T*_max_ = 0.746
26568 measured reflections
4674 independent reflections
3614 reflections with *I* > 2.0σ(*I*)
*R*_int_ = 0.035

#### Refinement

*R*[*F* ^2^ > 2σ(*F* ^2^)] = 0.040
*wR*(*F* ^2^) = 0.110
*S* = 1.05
4674 reflections
245 parameters
H-atom parameters constrained
Δρ_max_ = 0.16 e Å^−3^
Δρ_min_ = −0.22 e Å^−3^

### 

Data collection: *APEX2* (Bruker, 2007 ▶); cell refinement: *SAINT-Plus* (Bruker, 2007 ▶); data reduction: *SAINT-Plus*; program(s) used to solve structure: *SHELXS97* (Sheldrick, 2008 ▶); program(s) used to refine structure: *SHELXL97* (Sheldrick, 2008 ▶); molecular graphics: *SHELXTL* (Sheldrick, 2008 ▶); software used to prepare material for publication: *SHELXTL*.

## Supplementary Material

Crystal structure: contains datablocks Iloable, I. DOI: 10.1107/S1600536810054103/pv2365sup1.cif

Structure factors: contains datablocks I. DOI: 10.1107/S1600536810054103/pv2365Isup2.hkl

Additional supplementary materials:  crystallographic information; 3D view; checkCIF report

## Figures and Tables

**Table 1:** Hydrogen-bond geometry (Å, °)

*D*—H⋯*A*	*D*—H	H⋯*A*	*D*⋯*A*	*D*—H⋯*A*
C1—H1*A*⋯*Cg*1_C10-C15_^i^	0.96	2.98	3.781 (2)	142
C1—H1*C*⋯*Cg*2_C2-C7_^ii^	0.96	2.74	3.595 (2)	149

Symmetry codes: (i) 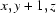; (ii) 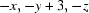.

**Fig. 1.**
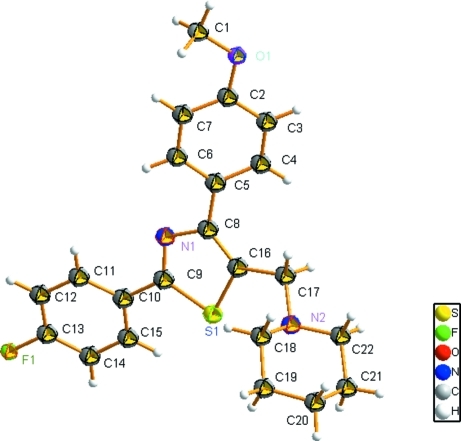
The structure of the compound (I) showing 50% probability displacement ellipsoid and the atom numbering scheme.

**Fig. 2.**
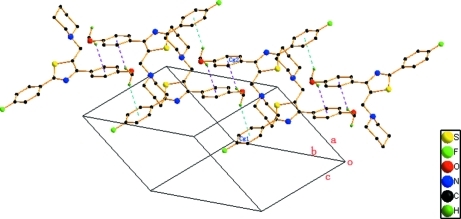
The three-dimensional structure of the title compound, formed through C—H···π interactions; the dashed lines represent C—H···π interactions.

## supplementary crystallographic information

### Comment

Thiazole derivatives have a varity of physiological effects, such as antiinflammatory (Guo *et al.*, 2006) and antimicrobial (Karegoudar *et al.*, 2008). Herein, we report the crystal structure of a new thiazole compound.

In the title compound (Fig. 1), the piperidine ring shows a chair confirmation. The thiazole fragment (S1/C9/N1/C8/C16) is essentially planar and it's mean plane makes dihedral angles of 29.2 (6) and 19.8 (1)°, with benzene rings C2—C7 and C10—C15, respectively, while the dihedral angle between the two phenyl rings is 17.0 (6)°.

The molecular packing is stabilized by C—H···π interactions (Fig. 2). The C1—H1*A*···*Cg1* distances are 3.781 (2) Å and C1—H1*C*···*Cg2* 3.595 (2) Å, respectively (*Cg* 1 and 2 are is the centroids of the aromatic rings C10–C15 and C2–C7, respectively. All the C—H···π interactions have been listed in Table 1 and presented in Fig. 2.

For related literature, see: Mitsutaka *et al.* (2006);

### Experimental

To a solution of piperidine (0.5 ml) in tetrahydrofuran (THF, 15 ml) was added a solution of 5-(bromomethyl)-2-(4-fluorophenyl)-4-(4-methoxyphenyl)thiazole (0.35 g, 0.92 mmol) in THF (10 ml), and the resulting mixture was stirred at 296 K for 1 h. The precipitate was filtered, the solvent was removed by rotary evaporation, the residue was dissolved in ethyl acetate, washed the organic phase with water and brine, dried over Na_2_SO_4_, filtered, and concentrated under reduced pressure to yield the title compound as a white powder (0.11 g, 32%). Crystals suitable for X-ray analysis were prepared by slow evaporation of a solution of the title compound in dichloromethane and methanol (*v*/*v* = 2:1) at room temperature in three days.

### Refinement

The H atoms were positioned in calculated positions with C—H = 0.93, 0.96 and 0.97 Å for aryl, methyl and methylene type H-atoms and were refined using a riding model, with *U*_iso_(H) = 1.5 *U*_eq_(C) for methyl H atoms and *U*_iso_ = 1.2*U*_eq_ for others.

### Crystal data

**Table:**

C_22_H_23_FN_2_OS	*Z* = 2
*M**_r_* = 382.48	*F*(000) = 404
Triclinic, *P*1	*D*_x_ = 1.289 Mg m^−^^3^
Hall symbol: -P 1	Mo *K*α radiation, λ = 0.71073 Å
*a* = 10.7565 (2) Å	Cell parameters from 7061 reflections
*b* = 10.8846 (2) Å	θ = 2.3–27.9°
*c* = 11.0179 (2) Å	µ = 0.19 mm^−^^1^
α = 67.035 (1)°	*T* = 296 K
β = 63.881 (1)°	Block, colourless
γ = 60.768 (1)°	0.26 × 0.26 × 0.24 mm
*V* = 985.16 (3) Å^3^	

### Data collection

**Table:**

Bruker APEXII CCD area-detector diffractometer	4674 independent reflections
Radiation source: fine-focus sealed tube	3614 reflections with *I* > 2.0σ(*I*)
graphite	*R*_int_ = 0.035
φ & ω scans	θ_max_ = 27.9°, θ_min_ = 2.1°
Absorption correction: multi-scan (*SADABS*; Bruker, 2007)	*h* = −14→14
*T*_min_ = 0.659, *T*_max_ = 0.746	*k* = −14→14
26568 measured reflections	*l* = −14→14

### Refinement

**Table:**

Refinement on *F*^2^	Primary atom site location: structure-invariant direct methods
Least-squares matrix: full	Secondary atom site location: difference Fourier map
*R*[*F*^2^ > 2σ(*F*^2^)] = 0.040	Hydrogen site location: inferred from neighbouring sites
*wR*(*F*^2^) = 0.110	H-atom parameters constrained
*S* = 1.05	*w* = 1/[σ^2^(*F*_o_^2^) + (0.0511*P*)^2^ + 0.1593*P*] where *P* = (*F*_o_^2^ + 2*F*_c_^2^)/3
4674 reflections	(Δ/σ)_max_ = 0.001
245 parameters	Δρ_max_ = 0.16 e Å^−^^3^
0 restraints	Δρ_min_ = −0.22 e Å^−^^3^

### Special details

**Table:**

Geometry. All e.s.d.'s (except the e.s.d. in the dihedral angle between two l.s. planes) are estimated using the full covariance matrix. The cell e.s.d.'s are taken into account individually in the estimation of e.s.d.'s in distances, angles and torsion angles; correlations between e.s.d.'s in cell parameters are only used when they are defined by crystal symmetry. An approximate (isotropic) treatment of cell e.s.d.'s is used for estimating e.s.d.'s involving l.s. planes.
Refinement. Refinement of *F*^2^ against ALL reflections. The weighted *R*-factor *wR* and goodness of fit *S* are based on *F*^2^, conventional *R*-factors *R* are based on *F*, with *F* set to zero for negative *F*^2^. The threshold expression of *F*^2^ > σ(*F*^2^) is used only for calculating *R*-factors(gt) *etc*. and is not relevant to the choice of reflections for refinement. *R*-factors based on *F*^2^ are statistically about twice as large as those based on *F*, and *R*- factors based on ALL data will be even larger.All hydrogen atoms were located in the calculated sites and included in the final refinement in the riding model approximation with displacement parameters derived from the parent atoms to which they were bonded.

### Fractional atomic coordinates and isotropic or equivalent isotropic displacement parameters (Å^2^)

**Table:**

	*x*	*y*	*z*	*U*_iso_*/*U*_eq_	
S1	0.33888 (5)	0.75870 (4)	0.04214 (4)	0.05238 (13)	
F1	0.59060 (15)	0.58770 (13)	−0.56262 (10)	0.0869 (4)	
O1	0.17330 (14)	1.58657 (12)	−0.00821 (13)	0.0640 (3)	
N1	0.36970 (13)	0.98214 (12)	−0.14604 (12)	0.0442 (3)	
C17	0.22778 (19)	0.90522 (17)	0.24743 (15)	0.0535 (4)	
H17A	0.3087	0.8712	0.2844	0.064*	
H17B	0.1614	1.0019	0.2622	0.064*	
C1	0.1157 (2)	1.70549 (18)	−0.1103 (2)	0.0711 (5)	
H1A	0.1916	1.7013	−0.1992	0.107*	
H1B	0.0863	1.7941	−0.0869	0.107*	
H1C	0.0302	1.7014	−0.1139	0.107*	
C2	0.20949 (16)	1.45111 (16)	−0.01833 (16)	0.0479 (3)	
C3	0.25260 (17)	1.33858 (17)	0.08932 (15)	0.0503 (3)	
H3	0.2557	1.3581	0.1627	0.060*	
C4	0.29100 (16)	1.19791 (16)	0.08885 (15)	0.0471 (3)	
H4	0.3210	1.1236	0.1614	0.056*	
C5	0.28547 (15)	1.16533 (15)	−0.01895 (14)	0.0428 (3)	
C6	0.24580 (18)	1.27941 (16)	−0.12758 (15)	0.0527 (4)	
H6	0.2446	1.2603	−0.2022	0.063*	
C7	0.20801 (19)	1.42074 (17)	−0.12818 (16)	0.0552 (4)	
H7	0.1817	1.4950	−0.2023	0.066*	
C8	0.31552 (15)	1.01781 (15)	−0.01896 (14)	0.0431 (3)	
C9	0.38848 (16)	0.84914 (15)	−0.12873 (14)	0.0438 (3)	
C10	0.44413 (15)	0.77917 (15)	−0.24329 (14)	0.0438 (3)	
C11	0.43754 (18)	0.86279 (17)	−0.37501 (15)	0.0524 (4)	
H11	0.3992	0.9629	−0.3906	0.063*	
C12	0.4874 (2)	0.79865 (19)	−0.48320 (16)	0.0595 (4)	
H12	0.4839	0.8544	−0.5715	0.071*	
C13	0.5419 (2)	0.65120 (19)	−0.45685 (16)	0.0584 (4)	
C14	0.5508 (2)	0.56486 (18)	−0.32943 (17)	0.0613 (4)	
H14	0.5890	0.4648	−0.3150	0.074*	
C15	0.50171 (18)	0.63000 (16)	−0.22271 (16)	0.0539 (4)	
H15	0.5073	0.5729	−0.1353	0.065*	
C16	0.29129 (17)	0.90992 (15)	0.09436 (14)	0.0470 (3)	
N2	0.14491 (14)	0.80983 (13)	0.32118 (12)	0.0468 (3)	
C18	0.00257 (19)	0.87436 (18)	0.29190 (16)	0.0571 (4)	
H18A	0.0214	0.9003	0.1924	0.068*	
H18B	−0.0614	0.9618	0.3268	0.068*	
C19	−0.0763 (2)	0.7702 (2)	0.35887 (19)	0.0665 (5)	
H19A	−0.0150	0.6851	0.3199	0.080*	
H19B	−0.1707	0.8155	0.3394	0.080*	
C20	−0.1053 (2)	0.7259 (2)	0.51460 (19)	0.0736 (5)	
H20A	−0.1780	0.8084	0.5555	0.088*	
H20B	−0.1464	0.6517	0.5548	0.088*	
C21	0.0389 (2)	0.6692 (2)	0.54608 (18)	0.0677 (5)	
H21A	0.1047	0.5777	0.5188	0.081*	
H21B	0.0171	0.6521	0.6452	0.081*	
C22	0.11816 (19)	0.77385 (18)	0.47077 (15)	0.0545 (4)	
H22A	0.0575	0.8615	0.5057	0.065*	
H22B	0.2131	0.7310	0.4888	0.065*	

### Atomic displacement parameters (Å^2^)

**Table:**

	*U*^11^	*U*^22^	*U*^33^	*U*^12^	*U*^13^	*U*^23^
S1	0.0689 (3)	0.0436 (2)	0.0398 (2)	−0.02424 (18)	−0.00998 (17)	−0.00904 (15)
F1	0.1297 (10)	0.0886 (8)	0.0513 (6)	−0.0570 (7)	−0.0035 (6)	−0.0326 (5)
O1	0.0744 (8)	0.0491 (6)	0.0779 (8)	−0.0251 (6)	−0.0247 (6)	−0.0200 (6)
N1	0.0470 (7)	0.0405 (6)	0.0400 (6)	−0.0140 (5)	−0.0111 (5)	−0.0106 (5)
C17	0.0668 (10)	0.0557 (9)	0.0420 (8)	−0.0297 (8)	−0.0106 (7)	−0.0136 (7)
C1	0.0652 (11)	0.0448 (9)	0.1013 (15)	−0.0165 (8)	−0.0290 (10)	−0.0170 (9)
C2	0.0450 (8)	0.0455 (8)	0.0561 (8)	−0.0192 (6)	−0.0107 (6)	−0.0172 (7)
C3	0.0542 (9)	0.0615 (9)	0.0483 (8)	−0.0292 (7)	−0.0146 (7)	−0.0171 (7)
C4	0.0491 (8)	0.0509 (8)	0.0458 (7)	−0.0220 (7)	−0.0186 (6)	−0.0069 (6)
C5	0.0397 (7)	0.0436 (7)	0.0421 (7)	−0.0150 (6)	−0.0086 (6)	−0.0126 (6)
C6	0.0681 (10)	0.0468 (8)	0.0445 (8)	−0.0185 (7)	−0.0199 (7)	−0.0134 (6)
C7	0.0687 (10)	0.0442 (8)	0.0498 (8)	−0.0172 (7)	−0.0239 (8)	−0.0072 (6)
C8	0.0427 (7)	0.0434 (7)	0.0406 (7)	−0.0150 (6)	−0.0111 (6)	−0.0109 (6)
C9	0.0446 (7)	0.0411 (7)	0.0401 (7)	−0.0141 (6)	−0.0106 (6)	−0.0097 (6)
C10	0.0440 (7)	0.0438 (7)	0.0405 (7)	−0.0172 (6)	−0.0083 (6)	−0.0115 (6)
C11	0.0627 (9)	0.0457 (8)	0.0460 (8)	−0.0214 (7)	−0.0145 (7)	−0.0096 (6)
C12	0.0754 (11)	0.0637 (10)	0.0414 (8)	−0.0331 (9)	−0.0155 (8)	−0.0082 (7)
C13	0.0689 (10)	0.0670 (10)	0.0452 (8)	−0.0350 (9)	−0.0023 (7)	−0.0243 (7)
C14	0.0758 (11)	0.0476 (9)	0.0529 (9)	−0.0231 (8)	−0.0083 (8)	−0.0181 (7)
C15	0.0654 (10)	0.0451 (8)	0.0428 (8)	−0.0184 (7)	−0.0124 (7)	−0.0106 (6)
C16	0.0524 (8)	0.0456 (8)	0.0412 (7)	−0.0198 (7)	−0.0102 (6)	−0.0118 (6)
N2	0.0540 (7)	0.0492 (7)	0.0366 (6)	−0.0219 (6)	−0.0106 (5)	−0.0102 (5)
C18	0.0599 (9)	0.0585 (9)	0.0471 (8)	−0.0178 (8)	−0.0179 (7)	−0.0113 (7)
C19	0.0593 (10)	0.0815 (12)	0.0655 (11)	−0.0297 (9)	−0.0174 (8)	−0.0216 (9)
C20	0.0693 (12)	0.0880 (14)	0.0620 (11)	−0.0436 (10)	−0.0075 (9)	−0.0123 (10)
C21	0.0751 (12)	0.0708 (11)	0.0480 (9)	−0.0359 (10)	−0.0140 (8)	0.0002 (8)
C22	0.0633 (9)	0.0591 (9)	0.0391 (7)	−0.0242 (8)	−0.0135 (7)	−0.0110 (7)

### Geometric parameters (Å, °)

**Table:**

S1—C16	1.7201 (15)	C10—C15	1.388 (2)
S1—C9	1.7264 (14)	C10—C11	1.392 (2)
F1—C13	1.3593 (17)	C11—C12	1.385 (2)
O1—C2	1.3665 (17)	C11—H11	0.9300
O1—C1	1.421 (2)	C12—C13	1.368 (2)
N1—C9	1.3060 (18)	C12—H12	0.9300
N1—C8	1.3912 (17)	C13—C14	1.365 (2)
C17—N2	1.4632 (19)	C14—C15	1.379 (2)
C17—C16	1.5054 (19)	C14—H14	0.9300
C17—H17A	0.9700	C15—H15	0.9300
C17—H17B	0.9700	N2—C22	1.4634 (18)
C1—H1A	0.9600	N2—C18	1.466 (2)
C1—H1B	0.9600	C18—C19	1.512 (2)
C1—H1C	0.9600	C18—H18A	0.9700
C2—C7	1.382 (2)	C18—H18B	0.9700
C2—C3	1.385 (2)	C19—C20	1.518 (3)
C3—C4	1.379 (2)	C19—H19A	0.9700
C3—H3	0.9300	C19—H19B	0.9700
C4—C5	1.3970 (19)	C20—C21	1.510 (3)
C4—H4	0.9300	C20—H20A	0.9700
C5—C6	1.389 (2)	C20—H20B	0.9700
C5—C8	1.4751 (19)	C21—C22	1.517 (2)
C6—C7	1.384 (2)	C21—H21A	0.9700
C6—H6	0.9300	C21—H21B	0.9700
C7—H7	0.9300	C22—H22A	0.9700
C8—C16	1.369 (2)	C22—H22B	0.9700
C9—C10	1.4734 (19)		
			
C16—S1—C9	89.65 (7)	C11—C12—H12	120.9
C2—O1—C1	117.72 (13)	F1—C13—C14	118.51 (15)
C9—N1—C8	110.92 (11)	F1—C13—C12	118.50 (15)
N2—C17—C16	111.16 (12)	C14—C13—C12	122.99 (14)
N2—C17—H17A	109.4	C13—C14—C15	118.20 (15)
C16—C17—H17A	109.4	C13—C14—H14	120.9
N2—C17—H17B	109.4	C15—C14—H14	120.9
C16—C17—H17B	109.4	C14—C15—C10	121.25 (15)
H17A—C17—H17B	108.0	C14—C15—H15	119.4
O1—C1—H1A	109.5	C10—C15—H15	119.4
O1—C1—H1B	109.5	C8—C16—C17	131.87 (13)
H1A—C1—H1B	109.5	C8—C16—S1	110.02 (10)
O1—C1—H1C	109.5	C17—C16—S1	118.06 (11)
H1A—C1—H1C	109.5	C17—N2—C22	111.27 (12)
H1B—C1—H1C	109.5	C17—N2—C18	111.22 (12)
O1—C2—C7	124.48 (14)	C22—N2—C18	110.21 (12)
O1—C2—C3	116.31 (13)	N2—C18—C19	110.86 (13)
C7—C2—C3	119.20 (14)	N2—C18—H18A	109.5
C4—C3—C2	120.72 (13)	C19—C18—H18A	109.5
C4—C3—H3	119.6	N2—C18—H18B	109.5
C2—C3—H3	119.6	C19—C18—H18B	109.5
C3—C4—C5	120.99 (13)	H18A—C18—H18B	108.1
C3—C4—H4	119.5	C18—C19—C20	110.67 (15)
C5—C4—H4	119.5	C18—C19—H19A	109.5
C6—C5—C4	117.27 (13)	C20—C19—H19A	109.5
C6—C5—C8	119.91 (12)	C18—C19—H19B	109.5
C4—C5—C8	122.80 (13)	C20—C19—H19B	109.5
C7—C6—C5	121.99 (14)	H19A—C19—H19B	108.1
C7—C6—H6	119.0	C21—C20—C19	109.97 (15)
C5—C6—H6	119.0	C21—C20—H20A	109.7
C2—C7—C6	119.77 (14)	C19—C20—H20A	109.7
C2—C7—H7	120.1	C21—C20—H20B	109.7
C6—C7—H7	120.1	C19—C20—H20B	109.7
C16—C8—N1	114.69 (12)	H20A—C20—H20B	108.2
C16—C8—C5	127.01 (13)	C20—C21—C22	111.94 (15)
N1—C8—C5	118.26 (12)	C20—C21—H21A	109.2
N1—C9—C10	124.09 (12)	C22—C21—H21A	109.2
N1—C9—S1	114.71 (10)	C20—C21—H21B	109.2
C10—C9—S1	121.19 (10)	C22—C21—H21B	109.2
C15—C10—C11	118.52 (13)	H21A—C21—H21B	107.9
C15—C10—C9	121.36 (13)	N2—C22—C21	111.09 (13)
C11—C10—C9	120.11 (13)	N2—C22—H22A	109.4
C12—C11—C10	120.77 (14)	C21—C22—H22A	109.4
C12—C11—H11	119.6	N2—C22—H22B	109.4
C10—C11—H11	119.6	C21—C22—H22B	109.4
C13—C12—C11	118.27 (15)	H22A—C22—H22B	108.0
C13—C12—H12	120.9		
			
C1—O1—C2—C7	6.7 (2)	C9—C10—C11—C12	−179.05 (14)
C1—O1—C2—C3	−174.43 (14)	C10—C11—C12—C13	0.6 (3)
O1—C2—C3—C4	179.86 (13)	C11—C12—C13—F1	179.83 (15)
C7—C2—C3—C4	−1.2 (2)	C11—C12—C13—C14	−0.8 (3)
C2—C3—C4—C5	−0.9 (2)	F1—C13—C14—C15	179.78 (15)
C3—C4—C5—C6	2.5 (2)	C12—C13—C14—C15	0.4 (3)
C3—C4—C5—C8	−175.70 (13)	C13—C14—C15—C10	0.2 (3)
C4—C5—C6—C7	−2.2 (2)	C11—C10—C15—C14	−0.4 (2)
C8—C5—C6—C7	176.13 (14)	C9—C10—C15—C14	178.63 (15)
O1—C2—C7—C6	−179.58 (14)	N1—C8—C16—C17	−176.66 (15)
C3—C2—C7—C6	1.6 (2)	C5—C8—C16—C17	1.1 (3)
C5—C6—C7—C2	0.1 (3)	N1—C8—C16—S1	0.64 (16)
C9—N1—C8—C16	−1.13 (18)	C5—C8—C16—S1	178.38 (12)
C9—N1—C8—C5	−179.08 (12)	N2—C17—C16—C8	146.91 (16)
C6—C5—C8—C16	−148.99 (16)	N2—C17—C16—S1	−30.21 (18)
C4—C5—C8—C16	29.2 (2)	C9—S1—C16—C8	−0.01 (12)
C6—C5—C8—N1	28.7 (2)	C9—S1—C16—C17	177.71 (13)
C4—C5—C8—N1	−153.12 (13)	C16—C17—N2—C22	164.61 (13)
C8—N1—C9—C10	179.82 (13)	C16—C17—N2—C18	−72.10 (16)
C8—N1—C9—S1	1.11 (16)	C17—N2—C18—C19	175.32 (13)
C16—S1—C9—N1	−0.66 (12)	C22—N2—C18—C19	−60.79 (17)
C16—S1—C9—C10	−179.41 (12)	N2—C18—C19—C20	58.33 (19)
N1—C9—C10—C15	161.52 (15)	C18—C19—C20—C21	−53.4 (2)
S1—C9—C10—C15	−19.8 (2)	C19—C20—C21—C22	52.2 (2)
N1—C9—C10—C11	−19.5 (2)	C17—N2—C22—C21	−177.25 (14)
S1—C9—C10—C11	159.13 (12)	C18—N2—C22—C21	58.89 (17)
C15—C10—C11—C12	0.0 (2)	C20—C21—C22—N2	−55.4 (2)

### Hydrogen-bond geometry (Å, °)

**Table:**

*D*—H···*A*	*D*—H	H···*A*	*D*···*A*	*D*—H···*A*
C1—H1A···Cg1_C10-C15_^i^	0.96	2.98	3.781 (2)	142
C1—H1C···Cg2_C2-C7_^ii^	0.96	2.74	3.595 (2)	149

Symmetry codes: (i) *x*, *y*+1, *z*; (ii) −*x*, −*y*+3, −*z*.

## References

[bb1] Bruker (2007). *APEX2*, *SAINT-Plus* and *SADABS* Bruker AXS Inc., Madison, Wisconsin, USA.

[bb2] Guo, C. B., Guo, Y. S., Guo, Z. R., Xiao, J. F., Chu, F. M. & Cheng, G. F. (2006). *Acta Chim. Sin* **64**, 1559–1564.

[bb3] Karegoudar, P., Karthikeyan, M. S., Prasad, D. J., Mahalinga, M., Holla, B. S. & Kumari, N. S. (2008). *Eur. J. Med. Chem.* **43**, 261–267. 10.1016/j.ejmech.2007.03.014 17540482

[bb4] Mitsutaka, K., Takayuki, H. & Midori, T. (2006). *Cryst. Growth Des.* **6**, 1945–1950.

[bb5] Reddy, K. A., Lohray, B. B., Bhushan, V., Bajji, A. C., Reddy, K. V., Reddy, P. R., Krishna, T. H., Rao, I. N. & Jajoo, H. K. (1999). *J. Med. Chem.* **42**, 1927–1940. 10.1021/jm980549x 10354401

[bb6] Sheldrick, G. M. (2008). *Acta Cryst.* A**64**, 112–122. 10.1107/S0108767307043930 18156677

[bb7] Takayuki, H., Kosuke, A., Midori, T. & Mitsutaka, K. (2009). *Cryst. Growth Des.* **9**, 3031–3035.

